# Seawater fish use an electrogenic boric acid transporter, Slc4a11A, for boric acid excretion by the kidney

**DOI:** 10.1016/j.jbc.2022.102740

**Published:** 2022-11-23

**Authors:** Akira Kato, Yuuri Kimura, Yukihiro Kurita, Min-Hwang Chang, Koji Kasai, Toru Fujiwara, Taku Hirata, Hiroyuki Doi, Shigehisa Hirose, Michael F. Romero

**Affiliations:** 1 School of Life Science and Technology, Tokyo Institute of Technology, Yokohama, Japan; 2 Graduate School of Bioscience and Biotechnology, Tokyo Institute of Technology, Yokohama, Japan; 3 Center for Biological Resources and Informatics, Tokyo Institute of Technology, Yokohama, Japan; 4 Department of Physiology and Biomedical Engineering, Mayo Clinic College of Medicine and Science, Rochester, Minnesota, USA; 5 Graduate School of Agricultural and Life Sciences, The University of Tokyo, Tokyo, Japan; 6 Nifrel, Osaka Aquarium Kaiyukan Co, Ltd, Osaka, Japan; 7 Nephrology & Hypertension, Mayo Clinic College of Medicine and Science, Rochester, Minnesota, USA; 8 O’Brien Urology Research Center, Mayo Clinic College of Medicine and Science, Rochester, Minnesota, USA

**Keywords:** electrogenic boric acid transporter, Slc4a11, electrophysiology, seawater acclimation, membrane transport, homeostasis, kidney, epithelial cell, AQP, aquaporin, BOR, boron transporter, BW, brackish water, cDNA, complementary DNA, eGFP, enhanced GFP, FW, fresh water, HEK293, human embryonic kidney 293 cell line, ICP-MS, inductively coupled plasma mass spectrometry, I–V, current–voltage, KLH, keyhole limpet hemocyanin, MDCK, Madin–Darby canine kidney, NMDG, *N*-methyl-d-glucamine, NIP, Nodulin26-like intrinsic membrane protein, Slc4, solute carrier family 4, SW, seawater

## Abstract

Boric acid is a vital micronutrient in animals; however, excess amounts are toxic to them. Little is known about whole-body boric acid homeostasis in animals. Seawater (SW) contains 0.4 mM boric acid, and since marine fish drink SW, their urinary system was used here as a model of the boric acid excretion system. We determined that the bladder urine of a euryhaline pufferfish (river pufferfish, *Takifugu obscurus*) acclimated to fresh water and SW contained 0.020 and 19 mM of boric acid, respectively (a 950-fold difference), indicating the presence of a powerful excretory renal system for boric acid. Slc4a11 is a potential animal homolog of the plant boron transporter BOR1; however, mammalian Slc4a11 mediates H^+^ (OH^−^) conductance but does not transport boric acid. We found that renal expression of the pufferfish paralog of *Slc4a11*, *Slc4a11A*, was markedly induced after transfer from fresh water to SW, and Slc4a11A was localized to the apical membrane of kidney tubules. When pufferfish Slc4a11A was expressed in *Xenopus* oocytes, exposure to media containing boric acid and a voltage clamp elicited whole-cell outward currents, a marked increase in pH_i_, and increased boron content. In addition, the activity of Slc4a11A was independent of extracellular Na^+^. These results indicate that pufferfish Slc4a11A is an electrogenic boric acid transporter that functions as a B(OH)_4_^−^ uniporter, B(OH)_3_-OH^−^ cotransporter, or B(OH)_3_/H^+^ exchanger. These observations suggest that Slc4a11A is involved in the kidney tubular secretion of boric acid in SW fish, probably induced by the negative membrane potential and low pH of urine.

Boron is essential to higher plants, primarily as a component of the polysaccharide cell wall matrix (1). In vertebrates, boric acid is a vital micronutrient that is required for embryogenesis in zebrafish and *Xenopus laevis* (2, 3). Boron plays beneficial roles in bone growth and maintenance, hormone function, brain function, arthritis amelioration, cancer risk reduction, and cell growth in mammals (4, 5, 6, 7, 8, 9); however, the biochemical mechanisms involved remain unclear. Nevertheless, high levels of boric acid are toxic to both plants and animals. In plants, boron toxicity reduces the growth and expansion of organs (10, 11), whereas in humans, gastrointestinal, cardiovascular, hepatic, kidney, and central nervous system effects, as well as dermatitis, erythema, and death have been observed in children and adults exposed to ≥84 mg boron/kg (5, 12, 13, 14, 15). The reported acute oral LD_50_ values in mice and rats are 600 and 460 to 900 mg boron/kg body weight, respectively (16, 17). The reported fish LC_50_ values are highly variable ranging from 3.63 to 1000 mg boron/l under various test conditions (18, 19), and concentrations of 100 to 300 mg boron/l can cause the death of various aquatic vertebrates (20).

Boron transporters (BORs) (21, 22) and boric acid channels (Nodulin26-like intrinsic membrane proteins [NIPs]) (23, 24) have been cloned from the model plants, *Arabidopsis thaliana* and *Oryza sativa*. BOR1, a homolog of the solute carrier family 4 (Slc4; bicarbonate transporters) (25), is considered a boron efflux transporter and is required for both efficient boron uptake (22) and tolerance to high boric acid concentrations (10, 26). Conversely, NIPs are homologous to aquaporins (AQPs) and aquaglyceroporins (water channels) (23, 24). In root cells, boric acid enters the root cytosol *via* NIP5;1 (boric acid uptake channel) in the soil solution. In the stele, boric acid is loaded into the xylem by BOR1 in the cytosol of root cells (27). The potential mammalian homolog of BOR1 is Slc4a11 (bicarbonate transporter-related protein, BTR1) (9, 28). Human SLC4A11 was initially characterized as a Na^+^-coupled borate cotransporter, NaBC1 (9); however, recent studies have shown that human and mouse Slc4a11 display H^+^/OH^−^ transport in both a Na^+^-independent and Na^+^-coupled mode but do not transport B(OH)_4_^−^ or HCO_3_^−^ (29, 30, 31, 32, 33, 34, 35, 36). In addition, human SLC4A11 mediates water and ammonium transport (32, 37, 38). *SLC4A11* mutations cause recessive congenital hereditary endothelial dystrophy 2, Fuchs’ dystrophy, and Harboyan syndrome (39, 40, 41, 42, 43, 44, 45, 46). *Slc4a11*^−/−^ mice exhibit abnormal auditory brain responses, vestibular-evoked potential waveforms (47), increased corneal thickness, and corneal edema (37, 47).

Although marine teleosts (bony fish) live in seawater (SW; ∼450 mM NaCl, ∼50 mM Mg^2+^, ∼30 mM SO4^2−^, 10 mM Ca^2+^, 10 mM K^+^, etc.), marine fish blood is hypoosmotic to SW. To avoid dehydration, marine fish drink large amounts of SW, absorb water and electrolytes in the intestine, and excrete excess electrolytes back into the SW *via* the gills (branchial chloride cells) (*e.g.*, Na^+^, Cl^−^, and K^+^) or into the urine *via* the kidneys (*e.g.*, Mg^2+^ and SO_4_^2−^) (48). In the kidney, the glomeruli of SW fish filter considerably less blood and produce much less primary urine than their fresh water (FW) counterparts, and tubular secretion largely contributes to urine production in SW fish (48, 49, 50). Their proximal tubules actively secrete fluids containing Mg^2+^, SO_4_^2−^, and Cl^−^ into the tubular lumen (51, 52). The collecting ducts may then actively reabsorb water along with Na^+^ and Cl^−^ to reduce urine volume, producing a relatively small volume of isotonic urine with high concentrations of Mg^2+^ and SO_4_^2−^. SW also contains ∼0.4 mM boric acid (∼4.5 mg boron/l). Thus, SW fish are exposed to a continuous influx of boric acid that must be excreted. To discover how SW fish excrete boric acid, we used two closely related pufferfish: river pufferfish (*Takifugu obscurus*, euryhaline) and tiger pufferfish (*Takifugu rubripes*, marine) (53), whose genome has been sequenced (54). By comparing the gene expression between FW- and SW-acclimated pufferfish, renal and intestinal ion transporters that are involved in HCO_3_^−^, Ca^2+^, Mg^2+^, and SO_4_^2−^ excretion for SW acclimation were identified (50, 55, 56, 57). By searching for ion transporters upregulated in SW, we found that an Slc4a11 paralog, Slc4a11A, had high renal expression and was upregulated during SW acclimation. Functional characterization (electrophysiology) and immunohistochemistry caused us to hypothesize that Slc4a11A is an electrogenic boric acid transporter that plays a central role in excess boric acid excretion at the apical membrane of marine teleost renal tubules. These observations suggest that SW fish excrete excessive boric acid (acquired from drinking SW) into the urine *via* Slc4a11A, thereby avoiding boric acid toxicity and simultaneously maintaining low plasma boric acid levels. To the best of our knowledge, this study is the first characterization of a boric acid excretion mechanism in an animal as well as the first demonstration of the presence of an electrogenic boric acid transporter.

## Results

### Boric acid excretion by pufferfish kidney in SW

Boric acid (B(OH)_3_, p*K*a = 9.24) is in equilibrium with borate (B(OH)_4_^–^) in aqueous solutions. Hereafter, the term “boric acid” refers to the combination of both forms, and the term “borate” refers specifically to molecular species B(OH)_4_^–^. When referring to B(OH)_3_, we specifically state “B(OH)_3_.” [Boron] was determined by analyzing trace elements derived from boric acid and all other compounds containing boron.

**Figure 1:**
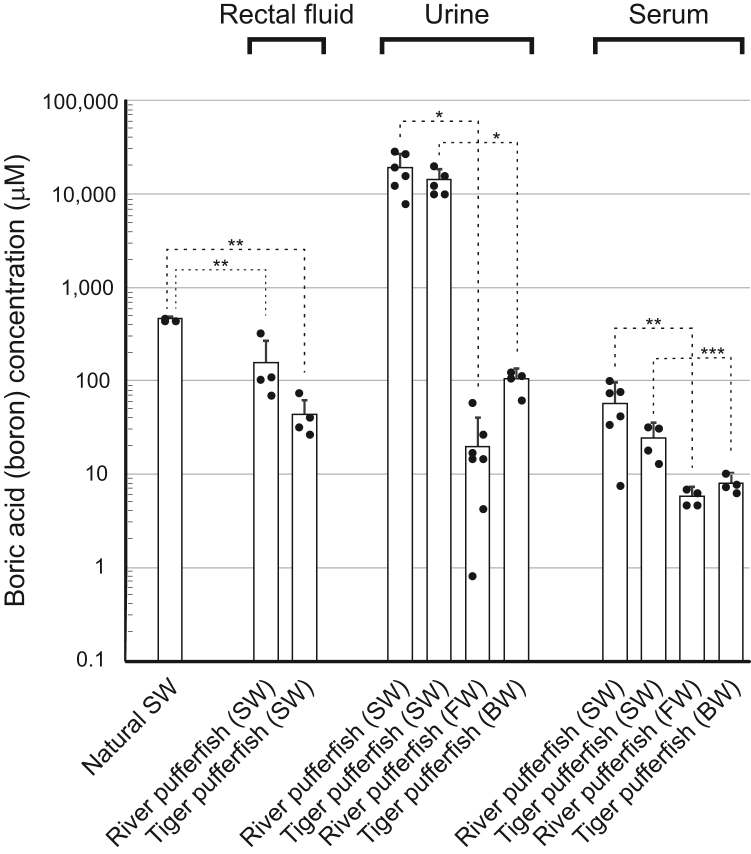
**Urinary boric acid excretion by pufferfish in seawater (SW).** Boric acid or boron concentrations of serum (n = 4–6), urine (n = 6–7), and rectal fluid (n = 4) of pufferfish acclimated to brackish water (BW), fresh water (FW), and natural SW are shown. *Dots* represent individual data. *Bar graphs* represent means ± SD. ∗*p* < 0.0001, ∗∗*p* < 0.0002, and ∗*p* < 0.05.

The natural SW used in this study contained 0.45 ± 0.02 mM boric acid (n = 3). The SW-acclimated river and tiger pufferfish contained intestinal or rectal fluids derived from ingested SW. The boric acid concentrations of all intestinal and rectal fluids were 0.15 ± 0.12 mM (n = 4) and 0.04 ± 0.02 mM (n = 4) in the SW-acclimated river and tiger pufferfish, respectively, and were significantly lower than that of SW (*p* < 0.0002) (Fig. 1), suggesting the presence of intestinal boric acid absorption by these fish. Intestinal and rectal fluids were not present in the intestines of FW-acclimated river pufferfish and brackish water (BW)–acclimated tiger pufferfish.

The serum [boron] of FW-acclimated river pufferfish was 6.0 ± 1.2 μM (n = 4, Fig. 1); [boron] refers to the total concentration of various boron forms expressed as boric acid equivalents. In SW, the serum [boron] increased to 57 ± 35 μM (n = 6), which was 9.5 times higher than that of river pufferfish in FW (*p* < 0.02). The serum [boron] of SW-acclimated tiger pufferfish was 24 ± 10 μM (n = 4), which was three times higher than that of tiger pufferfish in BW of 7.9 ± 1.7 μM (n = 4, *p* < 0.02). These results indicate that the [boric acid]_blood_ of pufferfish in SW was slightly higher than those of pufferfish in FW and BW, and that SW pufferfish maintained their [boric acid]_blood_ levels 7.9 to 19 times lower than that of marine [boric acid].

Bladder urine from river pufferfish in FW contained 20 ± 19 μM boric acid (n = 7). Interestingly, the bladder urine of river pufferfish in SW contained 19 ± 8.3 milli-M (n = 6) boric acid (*i.e.*, three orders of magnitude difference). In other words, the [boric acid]_urine_ of SW-acclimated river pufferfish was ∼950-fold greater than that of FW-acclimated fish (*p* < 0.0001), 330-fold greater than [boric acid]_blood_, and 41-fold greater than [boric acid]_SW_ (Fig. 1). Similar results were obtained with tiger pufferfish. Bladder urine of tiger pufferfish in SW contained 14 ± 4.5 mM boric acid (n = 5), which was ∼140-fold greater than that of BW-acclimated fish (*p* < 0.0001), 590-fold greater than [boric acid]_blood_, and 31-fold greater than [boric acid]_SW_. These results indicate that the SW pufferfish kidney has the ability to excrete and concentrate boric acid into the urine.

### Isolation and tissue distribution of Slc4a11A and Slc4a11B

**Figure 2:**
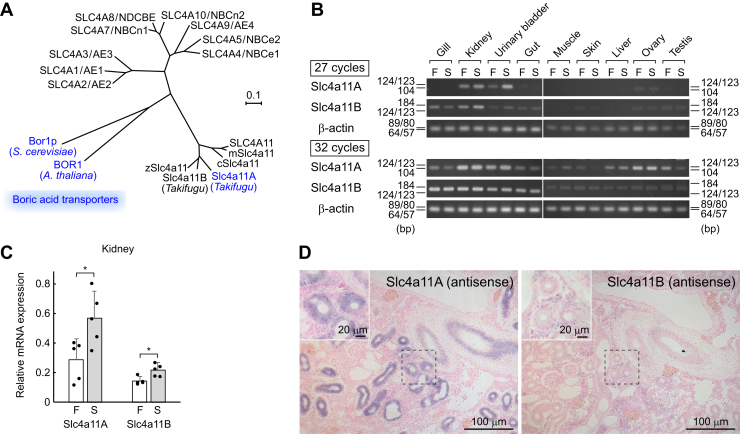
**Renal expression of Slc4a11A.***A*, phylogenetic tree of boric acid transporters in relation to the other human SLC4 family members. The boric acid transport activity of *Takifugu* Slc4a11A is shown in this study. The scale bar represents 0.1 amino acid substitution per site. *B*, tissue distribution of Slc4a11A and Slc4a11B. Semiquantitative RT–PCR was performed on various tissues of river pufferfish. Numbers indicate PCR cycles. Results from 27 PCR cycles show tissues with relatively high expression of the indicated genes, and those of 32 cycles show all tissues expressing the indicated genes from low to high levels (111, 162, 65). *C*, real-time PCR quantification of mRNAs for Slc4a11A and Slc4a11B in the kidneys of river pufferfish acclimated to FW and SW. Values are expressed relative to GAPDH. *Dots* represent individual data. *Bar graphs* represent means ± SD, n = 5. ∗*p* < 0.05. *D*, *in situ* hybridization of Slc4a11A and Slc4a11B in the kidney of river pufferfish in SW. Sense probes did not show labeling (data not shown). AE, anion exchanger; c, chicken; F, FW; NBC, Na^+^-HCO_3_^–^ cotransporter; NDCBE, Na^+^-driven Cl^–^/HCO_3_^–^ exchanger; S, SW; SLC4, solute carrier family 4; z, zebrafish.

**Figure 3:**
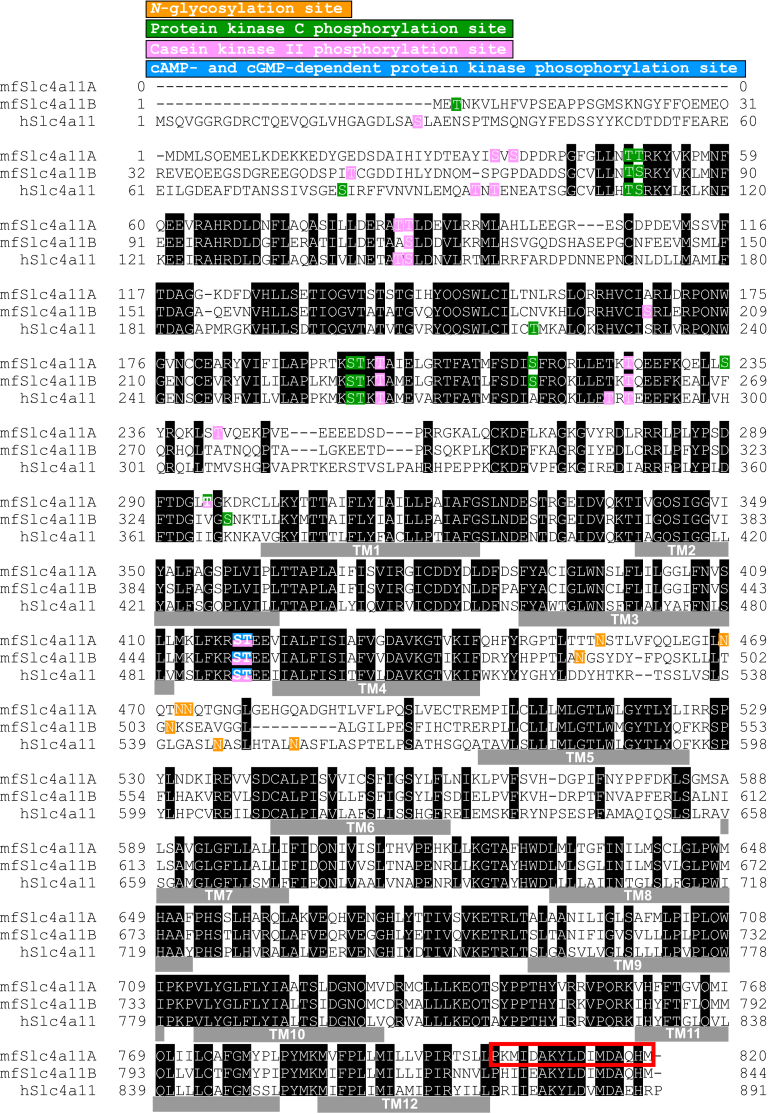
**Multiple alignment of amino acid sequences of Slc4a11 family.** The amino acid residues that are conserved among Slc4a11 family members are *shaded*. Transmembrane (TM) regions are indicated by *solid bars* labeled as TM1–TM12. The accession numbers of mfSlc4a11A, mfSlc4a11B, and hSLC4A11 are AB534190, AB534191, and NM_032034, respectively. h, human; mf, mefugu. *Red box* of mfSlc4a11A indicates the peptide antigen used to generate the mfSlc4a11A antibody.

We sought to investigate which transporter is involved in renal boric acid excretion into the urine. Two protein homologs of plant BORs were identified from the database of the *Takifugu* genome: Slc4a11A (820 amino acids) and Slc4a11B (844 amino acids). The phylogenetic relationships are shown in Figure 2*A*, and the multiple alignment of amino acid sequences is shown in Figure 3. Among the pufferfish tissues, Slc4a11A transcripts were notably high in the kidneys, whereas Slc4a11B transcripts were found at low levels in a broad range of tissues (Fig. 2*B*). Renal Slc4a11A expression in river pufferfish in SW was 1.6-fold higher than that of the fish in FW (Fig. 2*C*).

*In situ* hybridization histochemistry of kidney sections of SW-acclimated river pufferfish showed that Slc4a11A mRNA was highly concentrated in the renal tubules (Fig. 2*D*), whereas Slc4a11B mRNA was distributed in various renal cells, including a large number of nonepithelial cells, at low levels (Fig. 2*D*). This indicated that the major paralog in the renal tubules was Slc4a11A.

### Apical membrane localization of Slc4a11A in renal tubular cells

**Figure 4:**
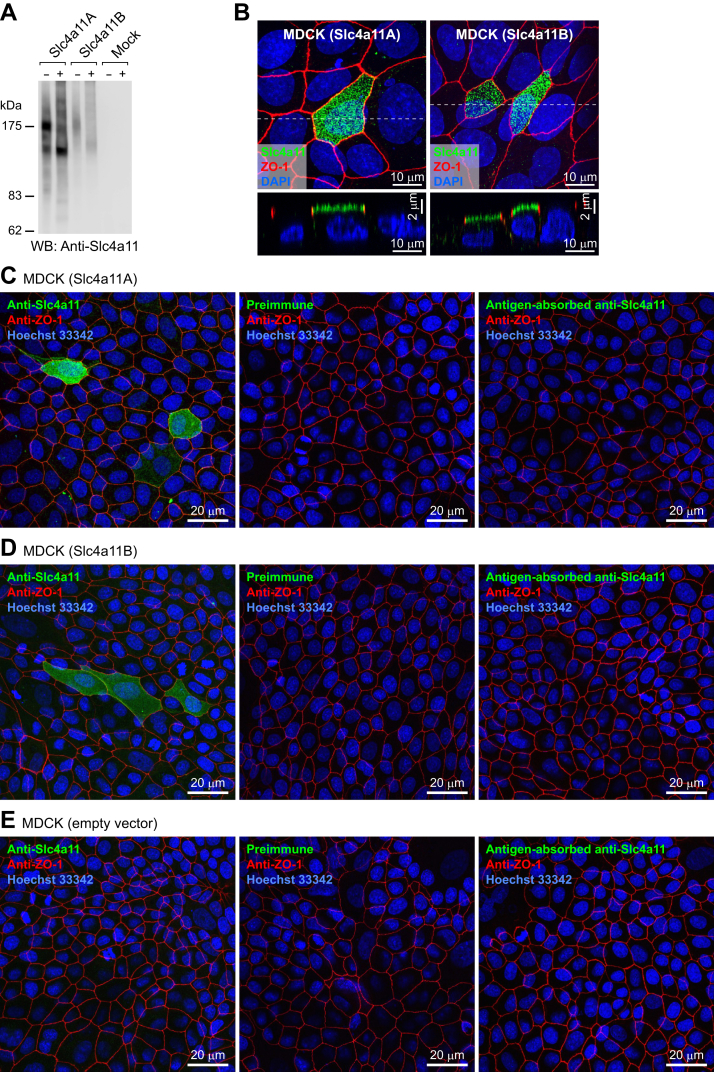
**Validation of the antibody against Slc4a11**. *A*, Western blot analysis of HEK293 cells transfected with pcDNA3 (mock), pcDNA3-Slc4a11A, or pcDNA3-Slc4a11B. The membrane fractions of the cells were incubated with (+) or without (–) glycosidases and analyzed using anti-Slc4a11 antiserum (*left*) and antigen-absorbed antiserum (*right*). *B*, polarized distribution of Slc4a11A and Slc4a11B in MDCK cells. Anti-Slc4a11 antiserum (*green*), anti-ZO-1 antibody (*red*), and Hoechst 33342 (*blue*) were used to stain MDCK cells transiently transfected with pcDNA3-Slc4a11A or pcDNA3-Slc4a11B. Confocal XY maximum projection image and XZ (vertical) sections are shown. *C-E*, anti-Slc4a11 antiserum (left panel), preimmune serum (center panel), or antigen-absorbed antiserum (right panel) (*green*) were used with anti-ZO-1 antibody (*red*) and Hoechst 33342 (*blue*) to stain MDCK cells transiently transfected with pcDNA3-Slc4a11A, pcDNA3-Slc4a11B, or pcDNA3 (mock). Confocal XY maximum projection images are shown.

To determine the subcellular localization of the Slc4a11A protein, a polyclonal antiserum was raised against a synthetic polypeptide based on the cytoplasmic Slc4a11A COOH terminus (Fig. 3, *red box*). The antibody recognized both Slc4a11A and Slc4a11B expressed in human embryonic kidney 293 (HEK293) cells (Fig. 4*A*). Incubation of the membrane fraction of HEK293 cells with glycosidases shifted the bands from ∼180 kDa to ∼120 kDa (Fig. 4*A*).

When Slc4a11A or Slc4a11B were expressed in Madin–Darby canine kidney (MDCK)–polarized epithelial cells, both were localized at the apical membrane (Fig. 4*B*). Immunoreactivity was not detected when preimmune serum or antigen-absorbed antiserum was used (Fig. 4*C*), showing that the antibody specifically recognized Slc4a11A or Slc4a11B expressed in MDCK cells.

**Figure 5:**
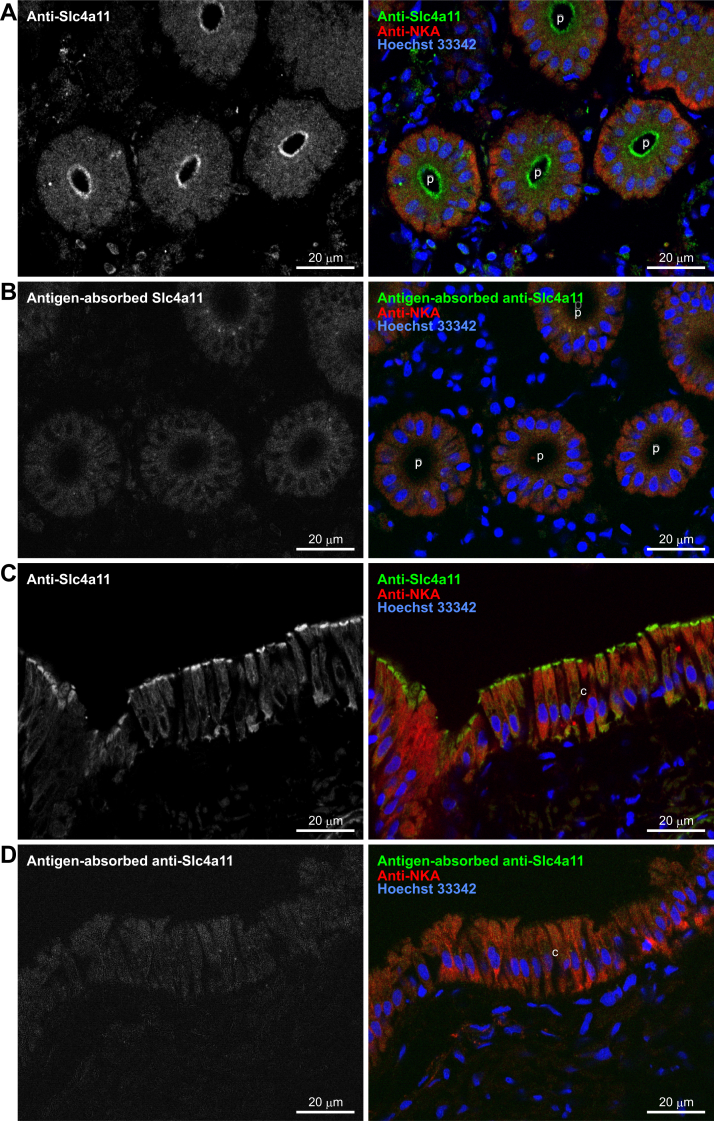
**Immunolocalization of Slc4a11 in renal tubules of SW-acclimated river pufferfish.** Serial frozen sections of mefugu kidney were stained with anti-Slc4a11 antiserum (*A* and *C*) or antigen-absorbed anti-Slc4a11 antiserum (*B* and *D*) (*green*), anti-Na^+^-K^+^-ATPase (NKA) antibody (*red*), and Hoechst (*blue*). Bars represent 20 μm. c, collecting duct; p, proximal tubule; Slc4, solute carrier family 4; SW, seawater.

We next stained the kidney sections of SW-acclimated river pufferfish with anti-Slc4a11 antibody. The nephrons of river pufferfish consist of a renal corpuscle, proximal tubule, distal tubule, and collecting duct (58). Previously, we identified these segments by the expression of molecular markers: the proximal tubule was identified by the presence of a brush border membrane and the absence of Na^+^–K^+^–2Cl^−^-cotransporter 2 (Nkcc2 or Slc12a1) and Na^+^–Cl^−^-cotransporter (Ncc or Slc12a3) expression; the distal tubule was identified by the absence of a brush border membrane and the presence of Nkcc2 expression; the collecting duct was identified by the absence of a brush border membrane and the presence of Ncc expression. In the same study, we also showed that all proximal tubules, distal tubules, and collecting ducts expressed Na^+^/K^+^-ATPase at high levels in the basolateral membrane, and immunostaining of Na^+^/K^+^-ATPase could visualize the difference in structure of the basolateral infoldings of the plasma membrane. Therefore, the segments can be distinguished by the shape of tubular cells and the immunostaining pattern of Na^+^/K^+^-ATPase. Proximal tubular cells are cuboidal epithelial cells with shallow basolateral infoldings, whereas distal tubular cells are cuboidal epithelial cells with deep basolateral infoldings, and collecting duct cells are columnar epithelial cells with deep basolateral infoldings (58). In the kidney sections of SW-acclimated river pufferfish, Slc4a11 was localized at the apical membranes of the proximal tubules and collecting ducts (Fig. 5) but not in the distal tubules (data not shown). Immunoreactivity was not detected when preimmune serum or antigen-absorbed antiserum was used (Fig. 5). This positive staining likely represents the Slc4a11A protein because the cells were almost identical to those identified by *in situ* hybridization histochemistry of Slc4a11A. Therefore, we focused on Slc4a11A for further functional characterization.

### Electrogenic boric acid transport activity of Slc4a11A expressed in *Xenopus* oocytes

**Figure 6:**
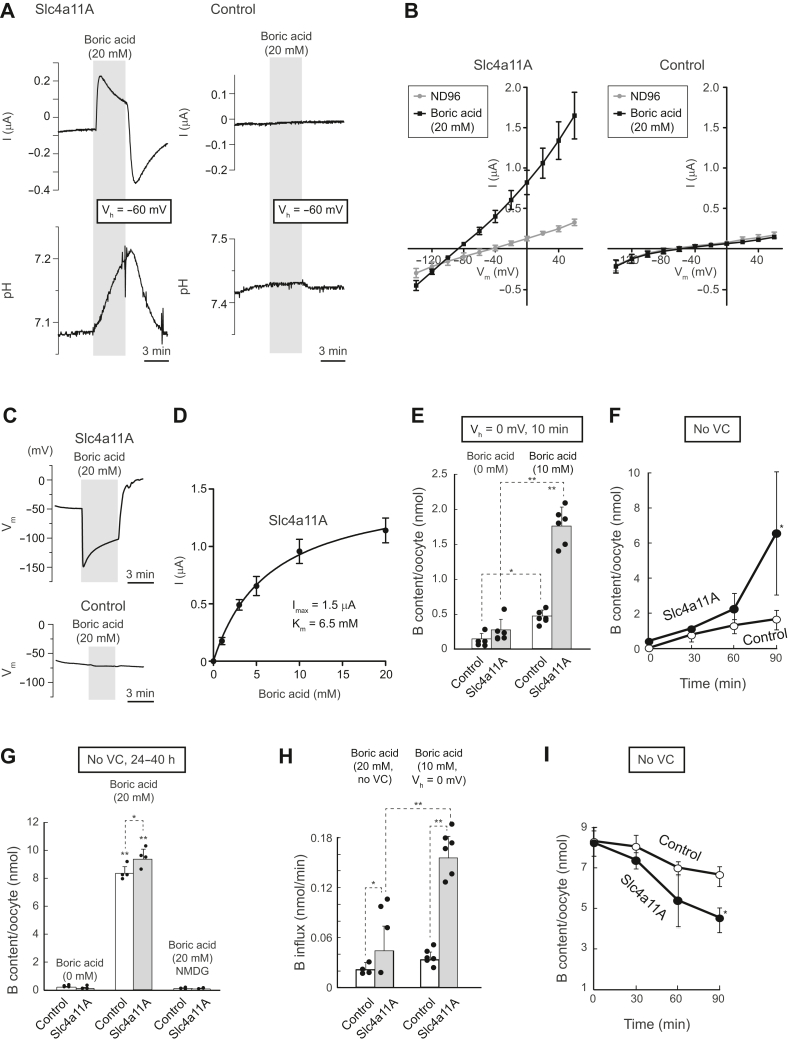
**Boric acid transport mediated by Slc4a11A.***A*, representative traces of boric acid–elicited currents and changes in intracellular pH of voltage-clamped oocytes (holding potential *V*_h_: −60 mV) injected with Slc4a11A or water (control). *B*, current–voltage (I–V) relationships of oocytes expressing Slc4a11A and control oocytes in the presence or the absence of 20 mM boric acid. Values are means ± SD, n = 5 to 8. *C*, representative traces of boric acid–elicited changes of membrane potential (*V*_m_) of oocytes injected with Slc4a11A and water (control). *D*, Michaelis–Menten curve fitted to boric acid–elicited currents of oocytes expressing Slc4a11A at +60 mV. Boric acid–elicited currents were measured by the addition of 1, 3, 5, 10, and 20 mM boric acid and were calculated as I_(boric acid)_ – I_(no boric acid)_. Maximum current (*I*_max_) and Michaelis–Menten constant (*K*_*m*_) are shown. Values are means ± SEM, n = 3. *E*, boric acid uptake by voltage-clamped oocytes. Slc4a11A oocytes and control oocytes were voltage clamped (*V*_h_: 0 mV) in ND96 containing 10 mM boric acid for 10 min, and the amount of boron in each oocyte was measured by ICP-MS. *Dots* represent individual data. *Bar graphs* represent means ± SD, n = 6. *F*, time course of boric acid uptake by unclamped oocytes. Oocytes were incubated in an ND96 medium containing 20 mM boric acid for 30, 60, and 90 min, and the amount of boron in each oocyte was measured by ICP-MS. Values are means ± SD, n = 4. *G*, boric acid uptake by unclamped oocytes. Oocytes were incubated in test solutions for 24 to 40 h, and the amount of boron in each oocyte was measured by ICP-MS. *Dots* represent individual data. Bar graphs represent means ± SD, n = 4. No VC, unclamped. *H*, rates of boric acid influx in voltage-clamped (*V*_h_: 0 mV) and unclamped oocytes. The rates were calculated from data shown in (*E* and *F*). *Dots* represent individual data. *Bar graphs* represent means ± SD, n = 4 to 6. *I*, time course of boric acid efflux by unclamped oocyte. Oocytes were incubated in an ND96 medium containing 20 mM boric acid for 24 h until saturated, followed by incubation in ND96 for 5, 10, and 20 min, and the amount of boron in each oocyte was measured by ICP-MS. Values are means ± SD, n = 4. ICP-MS, inductively coupled plasma mass spectrometry; Slc4, solute carrier family 4.

Slc4a11A-mediated boric acid movement across the plasma membrane was initially suggested as boric acid–elicited whole-cell currents and changes in pH_i_ in *Xenopus* oocytes by pH microelectrode analysis during *V*_m_ clamping (Fig. 6*A*). In Slc4a11A oocytes, but not in water-injected (control) oocytes, a significant positive outward I_boric acid_ (+0.27 ± 0.03 μA, n = 4; anion influx) was elicited by the addition of 20 mM boric acid in the bath solution, which was reversed (−0.46 ± 0.10 μA, n = 4; anion efflux) by its removal (Fig. 6*A*, *upper panel*). Exposure to 20 mM boric acid also caused a marked increase in pH_i_ (alkalinization: +62 ± 22 × 10^−5^ pH units/s, n = 3) (Fig. 6*A*, *lower panel*). Conversely, water-injected oocytes did not show significant pH_i_ changes when exposed to 20 mM boric acid (Fig. 6*A*, *right*).

The current–voltage (I–V) relationship is shown in Figure 6*B*. Exposure to boric acid medium elicited whole-cell currents and shifted the reversal potential (Fig. 6*B*). When the membrane potential (*V*_m_) was not clamped, boric acid medium elicited a hyperpolarization (Δ*V*_m_) in Slc4a11A oocytes (−85.0 ± 5.6 mV, n = 4) but not in control oocytes (−2.1 ± 0.3 mV, n = 4) (Fig. 6*C*). The boric acid–elicited currents were dose-dependently increased, and the Michaelis–Menten constant (*K*_*m*_) for Slc4a11A was 6.5 ± 0.6 mM (*V*_h_ = +60 mV, pH 7.5) for boric acid (Fig. 6*D*). The *I*_max_ was 1.5 ± 0.05 μA.

To directly measure Slc4a11A-mediated boric acid movement across the plasma membrane, the boron content of the oocytes was determined using inductively coupled plasma mass spectrometry (ICP-MS) (Fig. 6*E*). In the absence of boric acid in the medium, the whole boron content of the Slc4a11A and control oocytes was 0.27 ± 0.16 (n = 6) and 0.14 ± 0.09 (n = 5), respectively. After incubating oocytes in a medium containing 10 mM boric acid (*V*_h_ = 0 mV for 10 min), the whole boron content of the Slc4a11A oocyte (1.8 ± 0.26 nmol/cell) was significantly higher than that of the water-injected oocytes (0.47 ± 0.09 nmol/cell, *p* < 0.001, n = 6), indicating the presence of Slc4a11A-mediated boric acid movement across the plasma membrane. In the absence of a voltage clamp, a longer incubation time (∼90 min) was needed to detect a significant increase in the intracellular boron content (Fig. 6*F*). The intracellular boron content of both Slc4a11A and control oocytes was saturated after 24 h incubation in boric acid media (Fig. 6*G*). This result shows that the plasma membrane of control oocytes has weak permeability to boric acid. Boron accumulation was completely inhibited when the boric acid medium contained *N*-methyl-d-glucamine (NMDG) (Fig. 6*G*). This inhibition was observed with both Slc4a11A and control oocytes, likely because of the NMDG-chelating boric acid (59, 60). The boric acid flux rate per unit time in voltage-clamped Slc4a11A oocytes (*V*_h_ = 0 mV) was higher than that in unclamped Slc4a11A oocytes (Fig. 6*H*). Boric acid efflux was analyzed by placing boric acid–saturated oocytes in boric acid–free medium. Slc4a11A oocytes more quickly reduced intracellular boric acid than the control oocytes (Fig. 6*I*). These results showed that Slc4a11A is an electrogenic boric acid transporter and that its negative membrane potential inhibits the boric acid influx activity of Slc4a11A.

### Slc4a11A is a B(OH)_4_^−^ uniporter, a B(OH)_3_-OH^−^ cotransporter, or a B(OH)_3_/H^+^ exchanger

**Figure 7:**
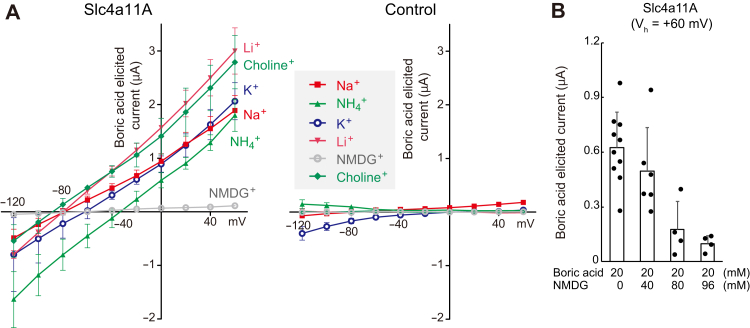
**Voltage-clamp analyses of Na**^**+**^**-independent electrogenic boric acid transport activity of Slc4a11A.***A*, current–voltage (I–V) relationships of Slc4a11A or water-injected (control) oocytes in a solution containing 20 mM boric acid and various cations. Boric acid–elicited currents calculated by subtraction are shown (n = 3 to 6). *B*, dose-dependent inhibition of boric acid transport activity of Slc4a11A by NMDG. Boric acid–elicited currents of Slc4a11A oocytes (holding potential *V*_h_: +60 mV) in the presence of various concentrations of NMDG are shown. Values are means ± SD (n = 4 to 10). NMDG, *N*-methyl-d-glucamine; Slc4, solute carrier family 4.

To determine whether Slc4a11A uses Na^+^-dependent cotransport, we replaced all Na^+^ in the media with various monovalent cations (choline^+^, NMDG^+^, Li^+^, K^+^, or NH_4_^+^) and compared the resulting boric acid–elicited currents in Slc4a11A-expressing *Xenopus* oocytes. Boric-elicited currents were also observed in Na^+^-free solutions containing choline^+^, Li^+^, K^+^, and NH_4_^+^ but not in those containing NMDG^+^ (Fig. 7*A*), indicating that Slc4a11A is a Na^+^-independent boric acid transporter. Figure 7*B* shows that 20 mM boric acid elicited whole-cell currents (*V*_h_ = +60 mV) in Slc4a11A oocytes in the presence of 0, 40, 80, or 96 mM NMDG, suggesting that the inhibition was observed only when NMDG was present at high concentrations, and the estimated IC_50_ value of NMDG in this condition was ∼67 mM.

**Figure 8:**
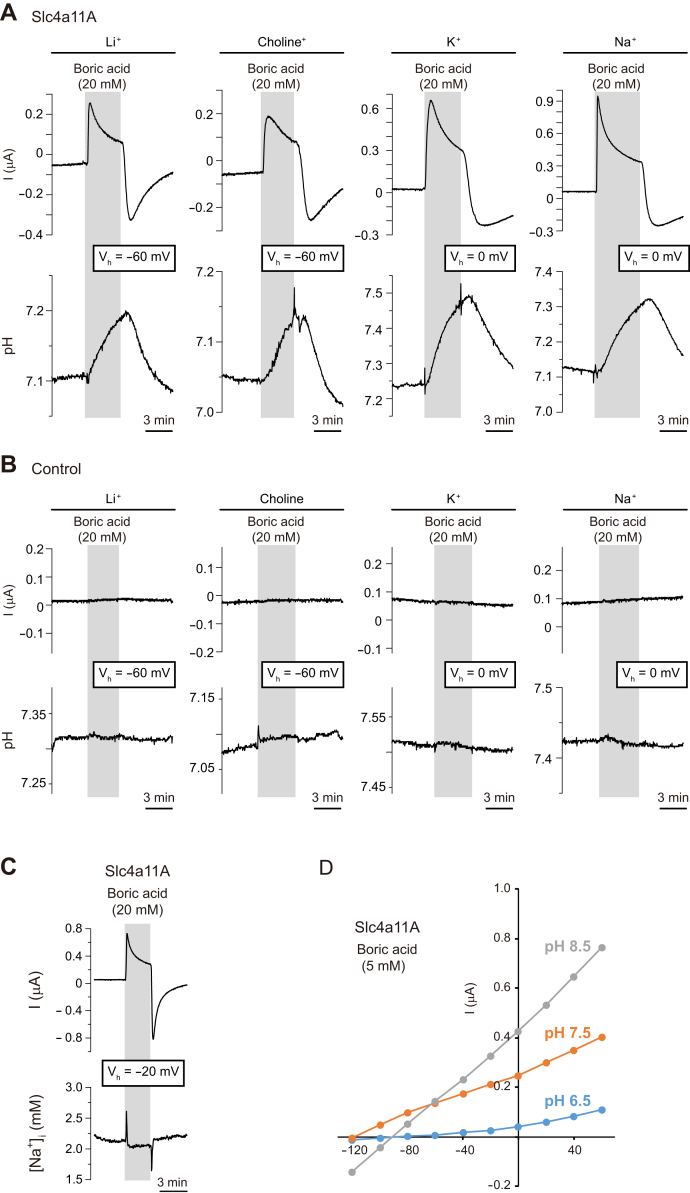
**Ion-selective microelectrode analysis during *V***_**m**_**clamping and pH dependence of Na**^**+**^**-independent electrogenic boric acid transport activity of Slc4a11A.** Representative traces of boric acid–elicited currents (holding potential *V*_h_: −60 or 0 mV) and changes in intracellular pH of Slc4a11A (*A*) and control (*B*) oocytes. Oocytes were analyzed in ND96 (indicated by Na^+^) or similar media in which Na^+^ was replaced with Li^+^, choline, or K^+^. *C*, representative traces of boric acid–elicited currents (holding potential, −20 mV) and intracellular [Na^+^] of Slc4a11A oocyte. *D*, current–voltage (I–V) relationship of 5 mM boric acid–elicited currents in various pH conditions. Values are means (n = 4 to 5). Slc4, solute carrier family 4.

Boric acid transport in Na^+^-free media was also confirmed by the marked increase in pH_i_ (Fig. 8*A*). Similar experiments were not performed with NH_4_^+^ because exposure to NH_4_^+^ alters pH_i_ and increases the buffering power of oocytes (61). Control oocytes did not show significant pH_i_ changes (Fig. 8*B*). The [Na^+^]_i_ of Slc4a11A oocytes did not change in solutions containing 20 mM boric acid (Fig. 8*C*). These results confirmed that Slc4a11A is a Na^+^-independent electrogenic boric acid transporter.

The ratio between the number for the net electric charge movement and that for the increased boron content was 1.1 (Table 1). To analyze the extracellular pH dependence of Slc4a11A activity, I–V relationships of Slc4a11A oocytes were analyzed in media containing 5 mM boric acid at pH levels of 6.5, 7.5, and 8.5 (Fig. 8*D*). The boric acid–elicited currents of Slc4a11A oocytes increased when the extracellular pH was increased. It is not clear whether this effect is due to the reduction of extracellular H^+^ (increase in extracellular OH^−^) or the altered equilibrium of boric acid, which increases the concentration of extracellular B(OH)_4_^−^. Taken together, these results indicate that Slc4a11A is a B(OH)_4_^−^ uniporter, B(OH)_3_-OH^−^ cotransporter, or B(OH)_3_/H^+^ exchanger.

**Table 1:** Comparison of electric charge movement and boron content of Slc4a11A oocytes

	Average ± SE
Electric charge movement (E_total_)	249 ± 17 μC
Electric charge movement by leak current (E_leak_)	95 μC
Net electric charge movement (E_boric acid_)	152 ± 15 μC
Number of net electric charge movements (A)	9.5 ± 0.9 × 10^20^
Boron content	1.77 ± 0.1 nmol
Boron content before incubation in medium containing boric acid	0.21 nmol
Increased boron content	1.56 ± 0.1 nmol
Number of increased boron contents (B)	9.0 ± 0.6 × 10^20^
Ratio (A/B)	1.1
Correlation coefficient	0.90
N	5
*p*	0.039

Oocytes were incubated in medium containing 10 mM boric acid at *V*_h_ of 0 mV for 10 min.

**Figure 9:**
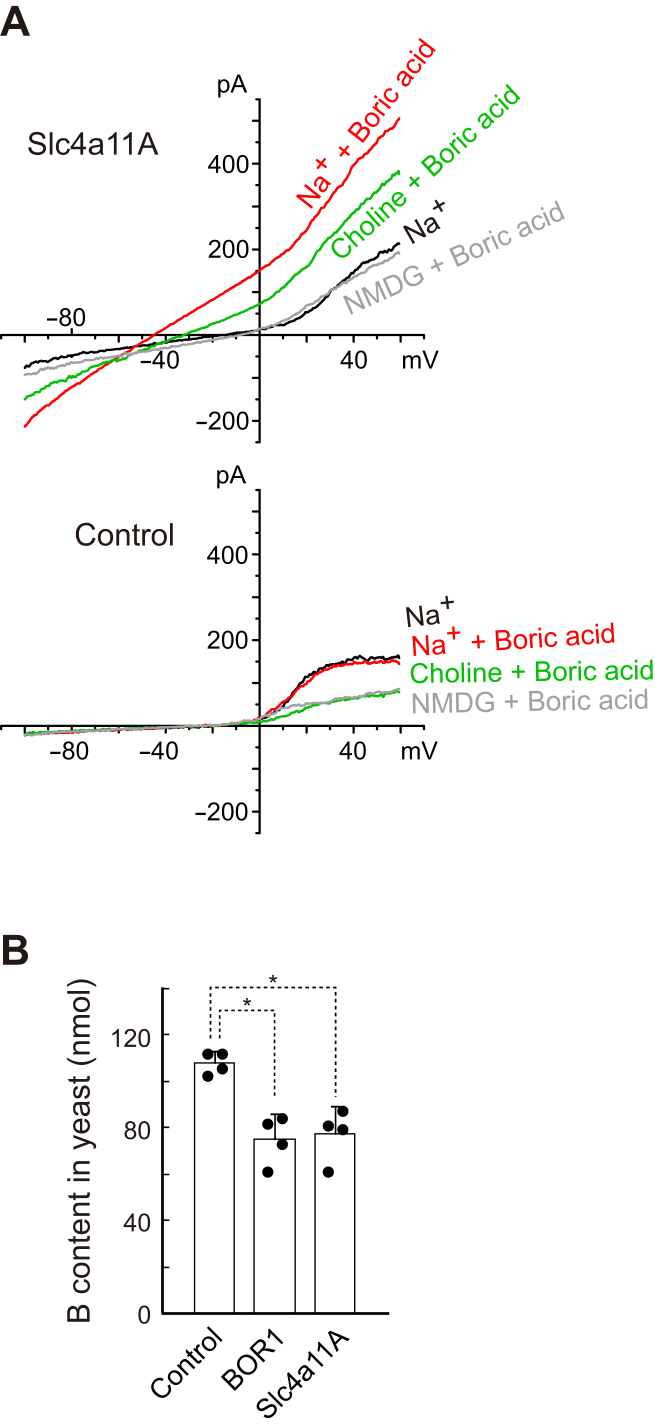
**Activity of Slc4a11A in HEK293 and yeast cells.***A*, the whole-cell current was measured in untransfected HEK293 cells (control) or HEK293 cells expressing eGFP-Slc4a11A. The cells were incubated in a solution containing 20 mM boric acid and 145 mM Na^+^ or Na^+^-free media in which all Na^+^ was replaced with choline or NMDG. *B*, concentration of boron in the yeast cells expressing Slc4a11s or AtBOR1. Cells were incubated in a medium containing 20 mM boric acid; boron concentration in these cells was measured by ICP-MS. *Dots* represent individual data. Values are means ± SD. ∗*p* < 0.002. eGFP, enhanced GFP; HEK293, human embryonic kidney 293 cell line; ICP-MS, inductively coupled plasma mass spectrometry; NMDG, *N*-methyl-d-glucamine; Slc4, solute carrier family 4.

To confirm whether the Na^+^-independent nature of Slc4a11A was specific to the *Xenopus* oocyte expression system, we measured the whole-cell currents of HEK293 cells expressing enhanced GFP (eGFP)-tagged Slc4a11A (Fig. 9*A*). I_boric acid_ was observed in HEK293 cells expressing eGFP-Slc4a11A and in Na^+^-free medium containing choline but not in that containing NMDG.

Yeast was used to compare the function of pufferfish Slc4a11A with that of the plant boron efflux transporter BOR1. A transformant of the *Saccharomyces cerevisiae* strain Y01169 carrying the yeast expression plasmid pYES-Slc4a11A was prepared, and its boron excretion was compared with that of the control strain lacking the endogenous BOR gene *bor1p*. When incubated with 20 mM boric acid, the transformant accumulated significantly lower levels of intracellular boron (*p* < 0.002, n = 4), which were similar to those of a BOR1 transformant (22) (Fig. 9*B*). This result indicates that Slc4a11A can export boric acid against a concentration gradient.

## Discussion

In this study, we found that pufferfish in SW, but not in FW or BW, excreted boric acid into their urine at a high rate. In other words, boric acid is a major component of marine fish urine. SW-acclimated river pufferfish contain high [boric acid]_urine_, whereas rectal fluid [boric acid] is quite low, demonstrating that the boric acid ingested from SW is absorbed by the gut and excreted by the kidney. The [boric acid]_urine_ of pufferfish in SW was 30 to 40 times greater than that of [boric acid]_SW_. This level of [boric acid]_urine_ is sufficient to account for the amount of SW-ingested boric acid, suggesting that the major route of boric acid excretion is through the urine.

Database mining and expression analyses, including *in situ* hybridization analyses, have shown that the major paralogs expressed in the renal tubules of river pufferfish are Slc4a11A. Moreover, *Slc4a11A* expression is upregulated in the kidney after SW acclimation, and the Slc4a11A protein is localized at the apical membrane of renal tubules, suggesting its crucial role in urine production during SW acclimation.

In humans, SLC4A11 was initially characterized as a Na^+^-coupled borate cotransporter (9); however, subsequent studies revealed that human SLC4A11 and mouse Slc4a11 mediate H^+^ (OH^−^) conductance but do not transport B(OH)_4_^−^ or HCO_3_^−^ (29, 30, 31, 32, 33, 34, 35, 36). In contrast to these studies on mammalian Slc4a11, we showed here that a fish paralog of Slc4a11, Slc4a11A, acts as an electrogenic boric acid transporter based on the following evidence: (1) dose-dependent boric acid elicited whole-cell currents (I_boric acid_) in *Xenopus* oocytes expressing Slc4a11A; (2) boric acid elicited a marked increase in pH_i_ in *Xenopus* oocytes expressing Slc4a11A; and (3) boric acid altered the intracellular boron content in *Xenopus* oocytes and a yeast strain expressing Slc4a11A. The difference in activity of Slc4a11 between mammals and marine teleosts raises a new question as to how Slc4a11 function evolved in vertebrate animals. Further studies on the activities of Slc4a11 orthologs and paralogs of various vertebrate species will clarify whether mammalian Slc4a11 lost boric acid transport activity or SW fish Slc4a11A acquired boric acid transport activity during the history of vertebrate evolution. In addition, further detailed studies on the activities of pufferfish Slc4a11A and Slc4a11B will clarify if pufferfish Slc4a11 paralogs have activity similar to that of mammalian Slc4a11 and if pufferfish Slc4a11A has activity other than electrogenic boric acid transport activity.

**Figure 10:**
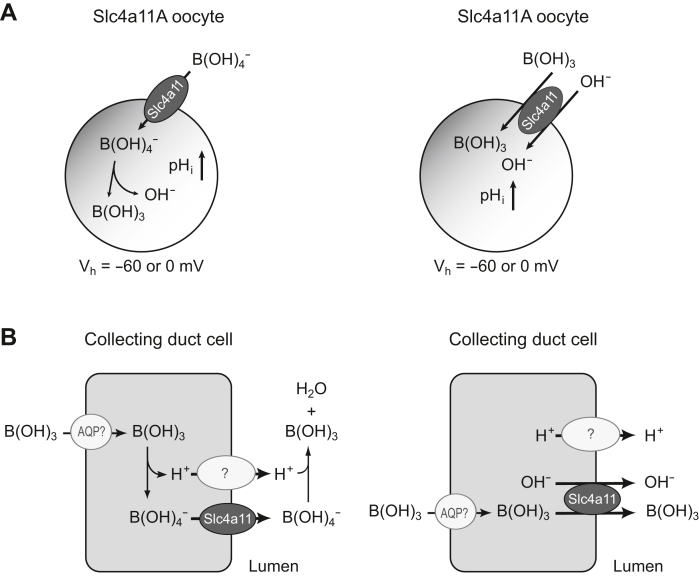
**Model of electrogenic boric acid transport activity of Slc4a11A.***A*, schematic representation of B(OH)_4_^−^ uniporter (*left*) or B(OH)_3_-OH^−^ cotransporter (*right*) activity of Slc4a11A in *Xenopus* oocytes. B(OH)_3_/H^+^ exchange activity is equivalent with B(OH)_3_-OH^−^ cotransport activity. *B*, hypothetical model of the epithelial secretion system for boric acid in the collecting duct cell of SW fish. Apical Slc4a11A mediates the B(OH)_4_^−^ uniport (*left*) or B(OH)_3_-OH^−^ cotransport (*right*), and the negative membrane potential and acidic pH of urine may be the driving forces for the luminal boric acid secretion. The activity of Slc4a11A may be coupled with apical H^+^-efflux systems, such as the Na^+^/H^+^ exchanger 3 (NHE3) or V-type H^+^-ATPase. Basolateral entry of boric acid from the plasma to the cytoplasm may be mediated by AQPs. AQP, aquaporin; Slc4a, solute carrier family 4; SW, seawater.

Electrophysiological experiments have demonstrated that Slc4a11A is an electrogenic boric acid transporter that is independent of Na^+^. Table 1 illustrates one anion influx or one cation efflux coupling to the influx of one boric acid molecule, indicating that Slc4a11A should be a B(OH)_4_^−^ uniporter, B(OH)_3_-OH^−^ cotransporter, or B(OH)_3_/H^+^ exchanger (Fig. 10*A*). These modes of action, in which negative membrane potential is the driving force, are advantageous for boric acid secretion against a concentration gradient and provide a thermodynamically favorable mechanism for the cellular efflux of boric acid. BOR1 is an efflux transporter in plants and yeast. Accordingly, BOR1 and Slc4a11A exhibit reduced [B]_i_ when expressed in yeast cells, suggesting that Slc4a11A (fish) and BOR1 (plants and yeast) secrete boric acid *via* a common mechanism.

The movement of boric acid through B(OH)_4_^−^ uniporter, B(OH)_3_-OH^−^ cotransporter, or B(OH)_3_/H^+^ exchanger will be determined by the (Equations (1), (2), (3)), respectively, when the stoichiometry of B(OH)_3_-OH^−^ cotransporter or B(OH)_3_/H^+^ exchanger are assumed to be 1:1,

(1) $$\Delta \mu_{B{\left(OH\right)}_{4}^{-}}=RT\cdot \ln\left(\frac{{\left[B{\left(OH\right)}_{4}^{-}\right]}_{i}}{{\left[B{\left(OH\right)}_{4}^{-}\right]}_{o}}\right)+\left(-1\right)F\Delta \Psi$$

(2) $$\Delta \mu_{B{\left(OH\right)}_{3}-OH^{-}}=\Delta \mu_{B{\left(OH\right)}_{3}}+\Delta \mu_{OH^{-}}=RT\cdot \ln\left(\frac{{\left[B{\left(OH\right)}_{3}\right]}_{i}}{{\left[B{\left(OH\right)}_{3}\right]}_{o}}\right)+RT\cdot \ln\left(\frac{{\left[OH^{-}\right]}_{i}}{{\left[OH^{-}\right]}_{o}}\right)+\left(-1\right)F\Delta \Psi =RT\cdot \ln\left(\frac{{\left[B{\left(OH\right)}_{3}\right]}_{i}\cdot {\left[OH^{-}\right]}_{i}}{{\left[B{\left(OH\right)}_{3}\right]}_{o}\cdot {\left[OH^{-}\right]}_{o}}\right)+\left(-1\right)F\Delta \Psi$$

(3) $$\Delta \mu_{B{\left(OH\right)}_{3}/H^{+}}=\Delta \mu_{B{\left(OH\right)}_{3}}-\Delta \mu_{H^{+}}=RT\cdot \ln\left(\frac{{\left[B{\left(OH\right)}_{3}\right]}_{i}}{{\left[B{\left(OH\right)}_{3}\right]}_{o}}\right)-\left\{RT\cdot \ln\left(\frac{{\left[H^{+}\right]}_{i}}{{\left[H^{+}\right]}_{o}}\right)+\left(+1\right)F\Delta \Psi \right\}=RT\cdot \ln\left(\frac{{\left[B{\left(OH\right)}_{3}\right]}_{i}\cdot {\left[H^{+}\right]}_{o}}{{\left[B{\left(OH\right)}_{3}\right]}_{o}\cdot {\left[H^{+}\right]}_{i}}\right)-\left(+1\right)F\Delta \Psi$$

where *R* is the gas constant, *T* is the absolute temperature, *F* is the Faraday constant, ln is the natural log, ΔΨ is the potential difference, and Δμ_solute_ is the electrochemical potential difference (Joules/mole). The dissociation constants of boric acid (*K*_a_) and water (*K*_w_) are:

$$K_{a}=\frac{\left[B{\left(OH\right)}_{4}^{-}\right]\cdot \left[H^{+}\right]}{\left[B{\left(OH\right)}_{3}\right]}$$

$$K_{w}=\left[H^{+}\right]\cdot \left[OH^{-}\right]$$

Therefore, the following equations hold:

$$\frac{{\left[B{\left(OH\right)}_{4}^{-}\right]}_{i}}{{\left[B{\left(OH\right)}_{4}^{-}\right]}_{o}}=\frac{{\left[B{\left(OH\right)}_{3}\right]}_{i}\cdot {\left[OH^{-}\right]}_{i}}{{\left[B{\left(OH\right)}_{3}\right]}_{o}\cdot {\left[OH^{-}\right]}_{o}}=\frac{{\left[B{\left(OH\right)}_{3}\right]}_{i}\cdot {\left[H^{+}\right]}_{o}}{{\left[B{\left(OH\right)}_{3}\right]}_{o}\cdot {\left[H^{+}\right]}_{i}}$$

$${\Delta \mu }_{B{\left(\text{OH}\right)}_{4}^{-}}={\Delta \mu }_{B{\left(\text{OH}\right)}_{3}-{\text{OH}}^{-}}={\Delta \mu }_{B{\left(\text{OH}\right)}_{3}/H^{+}}$$

When unclamped oocytes expressing Slc4a11A are incubated in ND96 solution containing 20 mM boric acid ([boric acid]_o_ = 20 mM) for several minutes, the whole boric acid content is assumed to be ∼0.1 nmol from Figure 6*H*. Whole oocyte volume is ∼0.7 μl, and when the volume of cytoplasm is assumed to be ∼10% of whole cell volume (62), [boric acid]_i_ can be calculated as ∼1.4 mM. pH_o_ is 7.5 and pH_i_ can be assumed to be ∼7.1. In these assumptions, using p*K*a value of boric acid, 9.24, [B(OH)_3_]_o_, [B(OH)_4_^−^]_o_, [B(OH)_3_]_i_, and [B(OH)_4_^−^]_i_ can be calculated as 19.6, 0.4, 1.39, and 0.01, respectively, and the ratio are calculated as:

$$\frac{{\left[B{\left(OH\right)}_{4}^{-}\right]}_{i}}{{\left[B{\left(OH\right)}_{4}^{-}\right]}_{o}}=\frac{{\left[B{\left(OH\right)}_{3}\right]}_{i}\cdot {\left[OH^{-}\right]}_{i}}{{\left[B{\left(OH\right)}_{3}\right]}_{o}\cdot {\left[OH^{-}\right]}_{o}}=\frac{{\left[B{\left(OH\right)}_{3}\right]}_{i}\cdot {\left[H^{+}\right]}_{o}}{{\left[B{\left(OH\right)}_{3}\right]}_{o}\cdot {\left[H^{+}\right]}_{i}}=0.03$$

The potential difference ΔΨ equivalent to this concentration difference is −89 mV at 18 °C. Therefore, this concentration difference is consistent with the hyperpolarization of −85 mV in Slc4a11A oocytes exposed to 20 mM boric acid solution when Slc4a11A acts as B(OH)_4_^−^ uniporter, B(OH)_3_-OH^−^ cotransporter, or B(OH)_3_/H^+^ exchanger.

Recently, we analyzed the boric acid transport activity of human AQPs and showed that AQP3, AQP7, AQP8, AQP9, and AQP10 act as boric acid transport systems, likely as B(OH)_3_ channels, based on the following evidence: (1) boric acid elicited a marked “decrease” in pH_i_ but did not elicit a change in membrane potential in *Xenopus* oocytes expressing these AQPs and (2) boric acid altered intracellular boron content in *Xenopus* oocytes expressing these AQPs (63). The results showed that the B(OH)_3_ transport activities of AQP3, AQP7, AQP8, AQP9, and AQP10 are electroneutral, suitable for the facilitated diffusion of B(OH)_3_ across the plasma membrane, and contrast with the electrogenic boric acid transport activity of Slc4a11A.

The resulting model for boric acid secretion in the kidneys of SW fish is shown in Figure 10*B*. In SW river pufferfish, the urine/plasma ratio of boric acid is ∼330; therefore, the kidney tubules secrete boric acid against the concentration gradient. We hypothesized how the apical electrogenic boric acid transporter Slc4a11A secretes boric acid into the urine and how the renal tubule concentrates boric acid. The negative membrane potential of renal tubular epithelial cells and the acidic pH of the urine of SW fish may induce the Slc4a11A-mediated boric acid secretion. Slc4A11A-mediated boric acid influx elicits a positive outward current, whereas Slc4A11A-mediated boric acid efflux elicits a negative outward current. Therefore, a negative membrane potential is beneficial for boric acid efflux from cells. Marine teleosts excrete acidic urine with a pH of 5.7 to 6.6 (64). If Slc4a11A is a B(OH)_3_-OH^−^ cotransporter or B(OH)_3_/H^+^ exchanger, the acidic pH of urine directly stimulates Slc4A11A-mediated boric acid efflux. Furthermore, if Slc4a11A is a B(OH)_4_^−^ uniporter, the acidic pH of urine is also beneficial for boric acid efflux, because secreted B(OH)_4_^−^ is converted to B(OH)_3_ by equilibrium in acidic urine and stimulates B(OH)_4_^−^ efflux by the B(OH)_4_^−^ uniporter. Two preconditions are necessary for this secretory model: (i) the basolateral membrane expresses the B(OH)_3_-permeable member of the AQP family that mediates the basolateral supply of boric acid for luminal secretion and (ii) the apical membrane does not express the B(OH)_3_-permeable member of the AQP family that can break the boric acid gradient across the apical membrane. Further analyses are required to determine the expression and intracellular localization of B(OH)_3_-permeable AQP(s) in the kidney tubules of SW fish.

## Experimental procedures

### Animals

Euryhaline pufferfish (river pufferfish, mefugu, *T. obscurus*) were purchased from local dealers in 2003 and 2011 and reared in tanks containing BW (3–14% diluted SW) (53, 56). They were transferred to FW tanks for 8 to 9 days (FW mefugu) and then to natural SW tanks for 8 to 9 days (SW mefugu). Marine pufferfish (tiger pufferfish, torafugu, *T. rubripes*) were purchased from a local dealer, reared in natural SW (53, 56), and then transferred to 150-l tanks containing BW (14% diluted SW) for 8 to 9 days (BW torafugu). The experimental animals were anesthetized by immersion in 0.1% ethyl m-aminobenzoate (MS-222; tricaine) neutralized with sodium bicarbonate. After humane killing by cervical transection, blood and urine were collected from the hepatic vein and urinary bladder, respectively, and the tissues were dissected. The animal protocols and procedures were approved by the Institutional Animal Care and Use Committee of Tokyo Institute of Technology (pufferfish and *Xenopus*) and Mayo Clinic (*Xenopus*).

### Quantitative determination of boron concentration by ICP-MS

Fish sera (200 μl), dried *Xenopus* oocytes, or dried yeast cells were digested with concentrated nitric acid in Teflon tubes (65), and the residues were dissolved in 0.08 M nitric acid containing 5 μg/l beryllium. Concentrations of ^10^B and ^11^B were measured by ICP-MS (Seiko Instruments) using beryllium as an internal standard, and the sum of the ^10^B and ^11^B concentrations was presented as the B concentration (22).

### Quantitative determination of boric acid concentrations using azomethine-H

Boric acid levels in urine, rectal fluid, and natural SW were determined using the azomethine-H method (66). Each sample (20 μl) was mixed with 40 μl of azomethine-H solution (10 mM azomethine-H and 57 mM l-ascorbic acid) and 40 μl of buffer-masking solution (6.5 mM ammonium acetate, 29 mM EDTA, 4.2 mM acetic acid, and 1.0 mM thioglycolic acid), incubated at 20 °C for 30 min, and measured for absorbance at 410 nm.

### Molecular cloning

Full-length complementary DNAs (cDNAs) for Slc4a11A (GenBank code: AB534190) and Slc4a11B (GenBank code: AB534191) were obtained by rapid amplification of cDNA ends (57) from the kidneys of SW-acclimated mefugu using the primers shown in Table 2.

**Table 2:** List of primers used for PCR amplification

Gene	Sequence	Remark
Slc4a11A	GGAGGAGGGCCGCGAGAGCTGC	Initial cloning (S)
	ACTTCAGCAGGCAACGATCTTTC	Initial cloning (AS)
	AAGTCCTTTCCTCCTGCGTCTGT	5ʹ RACE outer
	TCGCGGCCCTCCTCCAGCAGGTG	5ʹ RACE inner
	CACTTCTTCACAGGAGTGCAGATG	3ʹ RACE outer
	ACATCATGGATGCCCAACATATG	3ʹ RACE inner
	ACGAATGGATATGCTTTCGCAGG	ORF cloning (S)
	GCTCTGTAGTTACATATGTTGGGC	ORF cloning (AS)
	CCTCTGTGCGTTTGGGATGT	RT–PCR (S)
	GCGTCGATCATTTTAGGAAGCAG	RT–PCR (AS)
	CCTCTGTGCGTTTGGGATGT	Real-time PCR (S)
	GCGTCGATCATTTTAGGAAGCAG	Real-time PCR (AS)
	ATGGATATGCTTTCGCAG	CDS cloning (S)
	GCTCTGTAGTTACATATGTTGGGC	CDS cloning (AS)
Slc4a11B	GGAGACGACATCCACCTTTACG	Initial cloning (S)
	GGCAGCCGGCGGCACAGGTCCTC	Initial cloning (AS)
	AGCCACGACTGCTGGTACTG	5ʹ RACE outer
	TTCACATACTTCCGCGATGTGTT	5ʹ RACE inner
	TCACCTTCCTGCAGATGATGCAGC	3ʹ RACE outer
	GAGAGAGAGAAAGAGAAAAAGT	3ʹ RACE inner
	CACTCATCAGCACAGGATAACA	ORF cloning (S)
	TGATGACTGGACTGGGAGAACC	ORF cloning (AS)
	AGTGCCCCAGAGAAAGATCC	RT–PCR (S)
	ATGATGTGAGGAAGCACGTTG	RT–PCR (AS)
	AGTGCCCCAGAGAAAGATCC	Real-time PCR (S)
	ATGATGTGAGGAAGCACGTTG	Real-time PCR (AS)
β-actin	GCAAAACACCACACATTTCTCATAC	RT–PCR (S)
	ATGCCAATGAGTTGGTCGTCTA	RT–PCR (AS)
GAPDH	GGCCCAATGAAAGGCATTCT	Real-time PCR (S)
	TGGGTGTCGCCGTTGAA	Real-time PCR (AS)

Abbreviations: AS, antisense primer; CDS, CoDing Sequence; RACE, rapid amplification of cDNA ends; S, sense primer.

### Semiquantitative RT–PCR and real-time PCR

Semiquantitative RT–PCR was performed using total RNAs isolated from the tissues and primers shown in Table 2 as described previously (56, 57, 67). The relative renal expression of Slc4a11A and Slc4a11B was quantified by real-time PCR using the SYBR Green method, with GAPDH as a reference gene (56, 58). Significant differences at *p* < 0.05 were determined by a two-sample Student’s *t* test, assuming equal variance. Reproducibility was confirmed using two sets of experiments.

### Antibody production and absorption

Polyclonal antisera against Slc4a11s were generated by immunizing rabbits with a keyhole limpet hemocyanin (KLH)–conjugated peptide based on the C terminus of Slc4a11A (PKMIDAKYLDIMDAQHM) (Operon Biotechnologies). The antiserum (20 μl, 1:50 dilution) was absorbed with KLH (20 μg) in the presence or the absence of 27 μg of antigen peptide for 16 h at 4 °C and then for 2 h at 20 °C with continuous mixing.

### Expression of Slc4a11A and Slc4a11B in HEK293 and MDCK cells

Full-length cDNAs for Slc4a11A and Slc4a11B were subcloned into the pcDNA3 vector (Invitrogen). HEK293 and MDCK cells were transfected with pcDNA3-Slc4a11A, pcDNA3-Slc4a11B, or an empty vector using FuGENE6 (Roche), according to the manufacturer’s protocol. The cells were fixed, stained with antiserum, preimmune serum, or antigen-absorbed antiserum (1:1000 dilution), and then with the fluorescence-labeled secondary antibodies, phalloidin TRITC, and Hoechst 33342, as described previously (58). In the MDCK cells, anti–zona occludens 1 mouse monoclonal antibody (1:200 dilution; clone ZO1-1A12) was used to visualize tight junctions. Images were obtained with a confocal microscope (TCS-SPE; Leica) using the LAS AF software (Leica).

### Western blot analyses

Membrane fractions were collected as described previously (67, 68). Denatured protein samples were deglycosylated prior to blotting using a mixture of glycosidases (peptide-*N*-glycosidase and endo-α-*N*-acetylgalactosaminidase), according to the manufacturer’s instructions. The membrane proteins (1 μg) incubated with or without glycosidases were analyzed by Western blotting with anti-Slc4a11 antiserum or antigen-absorbed antiserum (1:10,000 dilution), as described previously (67, 68).

### Immunohistochemistry

The kidneys from SW mefugu were fixed and frozen in an optimum cutting temperature compound (Sakura Finetek). Sections (6 μm) were stained with the KLH-absorbed anti-Slc4a11 rabbit antiserum (1:20 dilution), anti-eel Na^+^-K^+^-ATPase rat antiserum, and fluorescence-labeled phalloidin as described previously (58). Fluorescence was detected as described previously.

### *In situ* hybridization

A 644-bp fragment of Slc4a11A cDNA (nucleotides 1028–1671) and a 240-bp fragment of Slc4a11B cDNA (nucleotides 1–240) were used to prepare digoxigenin-labeled sense and antisense riboprobes. Paraffin sections (4 μm) of the SW mefugu kidney were hybridized with the riboprobes that were visualized using alkaline phosphatase–conjugated antidigoxigenin antibodies and nitro blue tetrazolium/bromochloroindolyl phosphate substrates. Kernechtrot was used for nuclear counterstaining.

### Expression of Slc4a11A in *Xenopus* oocytes

Full-length cDNA for Slc4a11A was subcloned into a pGEMHE *Xenopus* expression vector and used for circular RNA synthesis (57, 69, 70). *X. laevis* oocytes were dissociated with collagenase (71) and injected with 50 nl of water or a solution containing circular RNA at 0.5 μg/μl (25 ng/oocyte). Oocytes were incubated at 16 °C in OR_3_ media (71) and studied 3 to 6 days after injection.

### Two-electrode voltage clamp analyses of *Xenopus* oocytes

Currents of oocytes were recorded as described previously at 18 °C (71). ND96 (96 mM NaCl, 2 mM KCl, 1 mM MgCl_2_, 1.8 mM CaCl_2_, and 5 mM Hepes, pH 7.5, ∼200 mosmol/kg) was used as the standard saline solution, and 70Cl-ND96 (ND96 containing 70 mM Cl^−^) was used as the standard bath solution (56, 57). To prepare 70Cl-ND96 containing 33 mM boric acid, NaCl (33 mM) was replaced with boric acid (33 mM), and the solution was titrated to pH 7.5 using NaOH. Test solutions with different boric acid concentrations were prepared by mixing 70Cl-ND96 with 70Cl-ND96 containing 33 mM boric acid followed by titration to pH 7.5. Boric acid–elicited currents were calculated as I_(boric acid)_-I_(no boric acid)_. The oocyte currents were recorded with an OC-725C voltage clamp (Warner Instruments) and Pulse software (HEKA) as previously described (72). The oocytes were clamped at a holding potential (*V*_h_) of −60 mV, and the current was constantly monitored and recorded at 1 Hz. The I–V protocols consisted of 200 ms steps from *V*_h_ in 20 mV steps between −160 and +60 mV. Data are expressed as the means ± SEM.

In 0-Na^+^ solutions, Na^+^ was replaced by choline, NMDG, K^+^, NH_4_^+^, or Li^+^ (73). To analyze the effect of NMDG on boric acid–elicited currents, 40, 80, and 96 mM Na^+^ of ND96 containing 20 mM boric acid were replaced with equal concentrations of NMDG. The IC_50_ was calculated with GraphPad Prism software (GraphPad Software, Inc). To analyze the effect of pH, the ND96 solution and that containing 5 mM boric acid were titrated to a pH of 6.5 and 7.5 using HCl and NaOH, respectively.

### Ion-selective microelectrode analysis of *Xenopus* oocytes

pH_i_ or [Na^+^]_i_ was measured as the difference between the pH or Na^+^ electrode and a KCl voltage electrode impaled into the oocyte, and the membrane potential (*V*_m_) was measured as the difference between the KCl microelectrode and an extracellular calomel at 18 °C (71). pH electrodes were calibrated using pH 6.0 and 8.0 buffer (pH) or 100 and 10 mM NaCl (Na^+^), followed by point calibration in ND96 (pH 7.50 or 96 mM) as described previously (71).

The ion-selective microelectrode analysis during *V*_m_ clamping was performed as previously described (74). One ion-selective electrode and two KCl electrodes were inserted into the oocyte, and the oocytes were clamped to a *V*_h_ of −60 or 0 mV in ND96 or 70Cl-ND96 solution after *V*_m_ and pH_i_ were stabilized. At steady state, the current and pH_i_ were monitored in response to the change in bath solutions.

### Boric acid uptake and efflux by *Xenopus* oocytes

For the boric acid uptake experiment with a voltage clamp, oocytes were voltage clamped at 0 mV and perfused with 70Cl-ND96 containing 10 mM boric acid for 10 min at 18 °C. For the experiment without a voltage clamp, oocytes were placed in 70Cl-ND96 containing 20 mM boric acid for set periods at 18 °C. For the boric acid efflux experiment without a voltage clamp, oocytes were placed in 70Cl-ND96 containing 20 mM boric acid for 24 h at 16 °C and then in ND96 for set periods at 18 °C. Each oocyte was washed with ND96 for several seconds and dried after the removal of the washing solution. The total boron content of each oocyte was quantified by ICP-MS, as described previously.

During the boric acid uptake experiment of oocytes using a voltage clamp, the current was recorded at 9 Hz and used to calculate the electrical charge movement (E_total_). The average leak current in 70Cl-ND96 was used to estimate the electrical charge movement using the leak current (E_leak_). The net electric charge movement of the oocytes elicited by 10 mM boric acid (E_boric acid_) was calculated by subtracting E_leak_ from E_total_. The number of net electric charge movements was calculated from the E_boric acid_ and elementary charge. The increase in boron content was calculated as the difference of the boron content before and after incubation in a medium containing boric acid. The increase in boron content was calculated using the Avogadro number. The ratio between the number of net electric charge movements and the number of increased boron content was calculated.

### Expression of Slc4a11A in HEK293 cells and measurement of its activity

Full-length cDNA for Slc4a11A was subcloned into a pEGFP-C vector, transiently transfected into HEK293 cells on poly-l-lysine–coated coverslips using FuGene6 and incubated for 24 to 48 h in culture medium. Currents of HEK293 cells were recorded using a HEKA EPC 10+ patch-clamp amplifier. The standard bath solution contained 140 mM NaCl, 5 mM KCl, 1 mM MgCl_2_, 10 mM Hepes (pH 7.5 with NaOH), 10 mM glucose, and 1 mM CaCl_2_. Extracellular solutions containing 20 mM boric acid were prepared by substituting 20 mM NaCl with 20 mM boric acid (pH 7.5, using NaOH). Na^+^-free bath solutions were prepared by substituting 140 mM NaCl with 140 mM choline Cl (pH 7.5, using choline base) or 140 mM NMDG (pH 7.5, using HCl).

### Expression of Slc4a11A in yeast cells and measurement of its activity

Full-length cDNAs for Slc4a11A cDNA was subcloned into the pYES2 yeast expression vector (Invitrogen) and expressed in the Y01169 *S. cerevisiae* strain as described previously (22). To measure boron concentration, cells in the midexponential phase were harvested, washed, and dried. The boron concentration was measured using ICP-MS, as described previously.

### Statistical analysis

Error bars represent SDs of the mean of at least three experiments. Statistical significance was calculated using an unpaired two-sided Student’s *t* test. Statistical significance for more than two groups was analyzed by one-way ANOVA followed by the Tukey–Kramer multiple comparisons test (GraphPad Prism 5).

## Data availability

The sequences reported in this article are available at the GenBank database with accession numbers AB534190 and AB534191. Other data are contained in the article. All information is available from the corresponding author upon reasonable request.

## Conflict of interest

The authors declare that they have no conflicts of interest with the contents of this article.
